# Identifying ‘hard-to-reach’ groups and strategies to engage them in biomedical research: perspectives from engagement practitioners in Southeast Asia

**DOI:** 10.12688/wellcomeopenres.15326.1

**Published:** 2019-06-26

**Authors:** Ha Nguyen Thanh, Phaik Yeong Cheah, Mary Chambers

**Affiliations:** 1 Oxford University Clinical Research Unit, HCMC, Vietnam; 2 Global Health Bioethics Network, Oxford, UK; 3 Centre for Tropical Medicine & Global Health, Nuffield Department of Medicine, Oxford, UK; 4 Mahidol Oxford Research Unit, Mahidol University, Bangkok, Thailand

**Keywords:** hard to reach, public engagement, community engagement, Southeast Asia

## Abstract

Public or community engagement (PE/CE) is an increasingly important component of biomedical research. However, PE/CE projects have been criticized for focusing on the ‘convenient sample’ populations that are more accessible and more likely to respond, thus missing out the less-socially visible groups. In January 2018, engagement practitioners from across Southeast Asia, attending a regional workshop, undertook a discussion about the ‘hard-to-reach’ populations in the region, and how PE projects can better engage them.  This paper is a summary of that discussion. After an initial brainstorming exercise the hard-to-reach populations identified by workshop participants were broadly categorised into three groups: urban poor, ethnic minority groups and children in rural primary schools. Delegates identified common characteristics of the populations and possible interventions to reach them. Notes of the discussions were used as data for the report. Four common issues that become barriers for engagement were identified: (1) financial instability; (2) mobility in residency and work; (3) discrimination and isolation; and (4) limitations in local resources. It is important to recognise that a group might be more disadvantaged by one factor than the others, but often these issues inter-relate to restrict outreach. In order to engage these populations, a tailor-made programme, that suits the local context, should be created. This can be done through four strategies that have the acronym ‘FIND’: (1) Formative research to improve understanding of the population; (2) Integrating into local life; (3) Networking with relevant stakeholders; and (4) Developing local resources.  Our discussion highlights the importance of a deep understanding of the local contexts in order to implement relevant and acceptable engagement projects. Findings from this report may be useful for planning public engagement projects in similar settings.

## Introduction

Public or community engagement (PE), often seen as a two-way interaction between experts and the public, is an increasingly important component in public health programmes and biomedical research, particularly for its important role in ethical conduct ( Cyril *et al.*, 2015; Marsh *et al.*, 2008; O'Mara-Eves *et al.*, 2015). However, one common critique is that PE activities or public health interventions are limited in focus. That is, despite the extensive meaning in the word ‘public’, these activities tend to reach a ‘convenient sample’ and thus miss out on a number of ‘hard-to-reach’ populations ( Cyril *et al.*, 2015; Guttman & Salmon, 2004; Silva *et al.*, 2013). The higher socio-economic groups are likely to be more responsive to recommended practice and achieve the desired outcomes, while engaging the hard to reach may not be effective ( Guttman & Salmon, 2004). The less socially visible groups, therefore, may have to be sacrificed for the need of maximized efficiency of public engagement interventions. Furthermore, failure to engage the disadvantaged groups may result in a generalization of the evidence of effective strategies used with the advantaged groups ( Cyril *et al.*, 2015; Silva *et al.*, 2013). There is also doubt over the authenticity of the claims that policy makers and public health practitioners understand the experience of disadvantaged population ( Silva *et al.*, 2013).

In the literature, these hard-to-reach groups are also called by different terms: the disadvantaged, the vulnerable, the community on the periphery ( Hanafin & Lynch, 2002), the underrepresented (Cortis, 2011), or the marginalized ( Silva *et al.*, 2013). They all acquire some similar characteristics: (i) being financially disadvantaged that may prevent them from accessing healthcare ( Cyril *et al.*, 2015; Freimuth & Mettger, 1990; Millar & Kilpatrick, 2005); (ii) belonging to different ethnic and racial groups that speak different languages and practice unique cultures, which hinders them from accessing mainstream health intervention and research ( Guttman & Salmon, 2004; Lalonde *et al.*, 1997); and (iii) having low level of literacy and limited skills to process health information and give consent ( Bonevski *et al.*, 2014; Freimuth & Mettger, 1990; Keselman *et al.*, 2015).

There is evidence that public and community engagement can be an effective approach to changing health behaviours and outcomes, as well as improving the quality of research for disadvantaged communities ( Attree *et al.*, 2011; Bonevski *et al.*, 2014; Cyril *et al.*, 2015; O'Mara-Eves *et al.*, 2013). However, most of these studies were conducted in high-income countries and there is a dearth of research discussing the impact of public engagement for hard-to-reach groups in low and middle-income nations. The living conditions and the needs of these groups in two settings can be very different, highlighting the need to explore the approach to reach the underrepresented population in developing settings.

Within this context, during a five-day regional conference on Public Engagement held in Ho Chi Minh City in January 2018, experienced public engagement practitioners from across Southeast Asia discussed how to identify and engage with hard-to-reach audiences in their countries including Vietnam, Thailand, Cambodia, Laos and Nepal.

## The workshop

The Oxford University Clinical Research Unit (OUCRU) and Mahidol Oxford Tropical Medicine Research Unit (MORU) are part of the Oxford Tropical Network and Nuffield Department of Medicine, University of Oxford. Their Public Engagement departments include staff in countries across the region including Cambodia, Indonesia, Laos, Myanmar, Nepal, Thailand and Vietnam. Their common aim is to engage local communities with biomedical research to inform and improve research and bring mutual benefit to communities and researchers. In January 2018 we convened a regional Public Engagement conference with 38 delegates from these sites, with a diversity of backgrounds and over a decade of engagement experience with a range of audiences – university students, school children, farmers, rural communities, patients and local health workers to name some. 

Over the course of the workshop, delegates were asked to identify audiences that they felt were hard to reach in each of their settings, and add them to a growing list. These were broadly categorized by a facilitator, with agreement of the group, into three groups including: urban poor, ethnic minority groups and children in rural primary schools. Participants were then invited to join one of three facilitated group to discuss: 1) common characteristics of the group; and 2) possible intervention to reach them, before reporting to the wider group for discussion. Minutes were taken by the first author to use as data for this report. The authors then consolidate ideas from the workshop to draw out the characteristics of hard-to-reach populations and solutions for improving outreach. Consent was given verbally from all workshop participants for their views to be represented in this manuscript.

## Why they are hard to reach

The workshop discussion is summarised in Table 1. Consideration of three discussion groups reflects four common issues of the hard-to-reach populations that become barriers to participation in engagement activities. These include: 1) financial instability; 2) mobility in residence and work; 3) discrimination and isolation; and 4) limitations in local resources. It also emerges that one group might be more disadvantaged in one factor than others, and these issues are often inter-related in restricting outreach.

**Table 1.** Summary of discussion on public engagement activities for hard-to-reach populations across Southeast Asia.

Groups	Characteristics	Why they are hard-to-reach	Suggested solutions to engage them
Urban poor	• Urban economic migrants • Migrant children in schools School drop-outs • Street vendors.	• Poor and so dedicate most time to work • Live away from home region and therefore unable to access health care or government benefits • Often mobile and move often	• Getting to know their concerns by making use of waiting time in hospitals/clinics to interview, putting Q&A box/board • Provide information from posters, leaflets during waiting time, radio shows • Collaborate with health stations/ public health clinics, schools, community organisations, factory owners
Ethnic minority groups	• A minority proportion of the total population with different cultures and languages • Live in closed communities, not mixing with other groups around them • Often live in rural areas, sometimes remote.	• Geographically isolation from health services • Language difference • Mistrust of ‘outsiders’ • Cultural and religious barriers to access mainstream intervention	• Need assessment to understand the community • Make use of local social activities, arts life-style and religious beliefs • Use art and theatre • Approach a community ‘gate keeper’: head of village/ teachers/ church/ local health workers/ local government/ volunteers • Let the community design activities with help from experts • Train local health staff
Rural school children	• Living in remote areas with lack of access to health care and health information • Study and also have to support parents with chores and agricultural work	• Geographical isolation • Lack of resources to do interventions • Lack of school’s and parent’s motivation for participation due to tight school curriculum and financial difficulties	• Use lunch time/breaks, one-off events • Integrate into school activities • Use their language, respect culture • Ask school teachers to approach parents • Train teachers; co-organise events with teachers • Offer incentives (furniture, library, stationery, more engagement)

### Financial instability

Corresponding with published literature, economic disadvantage was also reported in our workshop as one of the most important barriers to engagement with research and health care. With unstable financial conditions, the community members often have to devote most of their day to working and taking care of families, which leave them limited time to participate in medical interventions, research and PE activities. For example, industrial workers in the urban periphery follow very strict working schedules every day and have no time to learn about health. Agricultural work is the main source of income for ethnic minorities in Vietnam central highlands and on the Thai-Myanmar border. Adults, often taking their children with them, travel to the farms that may be far from their homes, from early morning until late afternoon, and so they are missed out from many health initiatives that take place within the commune during work hours. Rural school children have to spend more of their time outside of school hours helping parents with house chores or farming, compared to their urban peers. Parents may also be more reluctant to approve their children’s participation in extra activities outside school, and may be unable to be flexible with transportation to and from engagement events.

### Mobility in residence and work

All three discussion groups emphasized that the mobility in residence and workplace of these hard-to-reach populations is a significant challenge for local authorities and any social organizations wishing to monitor or engage with them. This is particularly problematic in Vietnam, where government benefits including both housing and healthcare are based on residency registration. Urban migrants or ethnic women moving between communes for marriage and work may fall through the net of government support, especially when this movement is not reported to the local authority. For example, recent neonatal tetanus cases have been identified in some ethnic communities in Dak Lak province, Vietnam. Investigations reveal that local health providers are unaware of women moving into their area and thus are unable to encourage them for maternal vaccination when they become pregnant. While public health programmes and research tend to involve the community through a structural and organizational approach, out-of-system populations in urban cities such as roaming street vendors and children who have dropped out of school are likely to be missed.

### Social discrimination and isolation

Differences in culture, language and lifestyles of the hard-to-reach groups often create barriers against their participation in mainstream interventions and biomedical research. This is a particular issue in the urban poor and ethnic communities. Migrant children often suffer from discrimination for their origins, accents and inferior living conditions, which makes them vulnerable to being bullied by peers at schools. As a result, they skip classes or even drop out completely, and are subsequently excluded from school engagement activities.

It is important to note that the issue of isolation in ethnic communities from mainstream interventions comes from structural, social and individual levels. On one hand, ethnic communities are reported to suffer from discrimination of healthcare staff in treatment and attitudes. On the other hand, there are ethnic groups that actively refuse to be involved with modern medicine and health intervention as they have long-established practices of using traditional medicine or it contradicts their religious beliefs. Such social gaps between communities and experts can be exacerbated by the difference in language and cultural practice. Ethnic languages may not be widely spoken by health staff, and may not be available in written form, making it difficult for researchers or healthcare staff to share health knowledge. Workshop delegates agreed that in their experience, minority groups are also more hesitant to participate in research and are reserved in providing information. Isolation may also be physical. Many ethnic minority communities live in inaccessible areas, such as the Karen minority in Thailand and Kravet community in Cambodia who live in mountainous areas, making them geographically excluded from mainstream healthcare and social initiatives.

### Limitations in local resources

Another barrier to widening public outreach that our workshop participants highlighted is the limitations in both local facilities and human resources in order to facilitate necessary changes. In rural or remote areas, lack of even basic resources such as electricity can pose difficulties in holding events. In rural schools in Ben Tre, Vietnam, the school is often only equipped with a few posters and simple teaching tools, with almost no equipment or laboratory facilities for conducting science experiments. While visual methods have increasingly been utilized to attract attention and improve learning for populations with limited health literacy, equipment such as projectors or televisions are very limited in remote areas. Thus, the use of media like PowerPoint presentations, videos and films, that are regarded fundamental in communication in urban areas, can be difficult for rural and ethnic communities.

Human resources are also inadequate in both numbers and skills. In remote areas, with ethnic minority populations, the number of health staff is often very limited compared to the amount of work they have, making it impossible for them to monitor and ensure full coverage in the local area. In Vietnam, medical experts such as doctors or the head of local clinics come from the dominant ethnic group and often find it difficult to communicate with the local minority ethnic community. Meanwhile, ethnic health staff may have limited medical expertise, making them unconfident and hesitant to deliver health-related participatory activities within their own community. Inadequate skills, knowledge and experience to use a more active and engaging approach in schools is also seen in rural school teachers. Schools may be reluctant to include more participatory or extracurricular activities as the academic curriculum is heavy. Much of the time teachers have a second job to supplement their meagre teaching salary.

## The interplay of four issues

These four issues are interconnected in restricting each group in participating in engagement and health programmes. For example, poverty is a motivation for people to migrate into the city for better employment opportunities. Many people from ethnic communities are moving to the city, and thus become the urban poor that suffer from discrimination and difficult living conditions. The lack of communication skills may separate the doctors from the ethnic communities, expanding the social gaps between practitioners and local residents. Such interplay reflects a need that intervention should employ a comprehensive approach that addresses multiple issues at the same time.

## How to reach the hard to reach

As reported in published literature, the delegates agreed that to reach these hard-to-reach groups, it is important to create a programme tailored to the needs and life contexts of the target population ( Cyril *et al.*, 2015; Guttman & Salmon, 2004; O'Mara-Eves *et al.*, 2013). Solutions proposed in the workshop discussions reflect four common strategies to tackle these issues, which can be abbreviated by the word ‘FIND’: 1) Formative research to meaningfully explore the population; 2) Integrating into local life; 3) Networking with relevant stakeholders; and 4) Developing local resources. As factors that influence outreach are interrelated to each other, each strategy can be used to address more than one difficulty.

### Formative research to meaningfully explore the population

The initial strategy that all three discussion groups agree on is the need to truly understand the local context. Any formative research should achieve three findings: (i) understanding the local life, especially specific issues that make a community hard to reach; (ii) identifying their particular needs; and (iii) mapping different stakeholders that play a key role in health promotion and engagement programmes. To avoid the superficial design of what is called ‘culturally appropriate’, public engagement practitioners, instead of just dwelling on recorded literature, are encouraged to directly explore the community. This is particularly important in understanding the ethnic communities. The formative research for community engagement to support for the Targeted Malaria Elimination study in the Lao Theung ethnic groups in Laos is a relevant example. With prior research into the beliefs, misconceptions, and misunderstanding of the community about malaria prevention and treatment, the PE team in the study were able to conduct activities to improve the community’s knowledge and awareness, which subsequently contributed to the tailored intervention study for malaria in the local area ( Adhikari *et al.*, 2017). Also, it is important to recognize that while we can generalize that the majority of ethnic minority peoples across Southeast Asia are disadvantaged when it comes to accessing services, some groups are more so. For example, in Vietnam, unpublished work by OUCRU reveals that the Ede in the central highlands and S’tieng in the south tend to be fairly well integrated into rural society and many speak Vietnamese, whilst other groups such as Mnong and H’mong in the central highlands remain separated culturally and linguistically. Therefore, formative research questions need to be very specific about the target population.

Common data collection methods include surveys, interviews and group discussions of relevant stakeholders but the approach needs to be flexible and foreground the customs of the target group. For a population that is significantly different in culture, language and lifestyle from the majority, it is suggested that public engagement practitioners should consider spending time living in the community as the ethnic minorities can be quite reserved in talking to the ‘outsiders’. For populations with low literacy, data collection can be participatory. In Vietnam, the Health in the Backyard engagement project encouraged ethnic farmers take photos of their husbandry activities and tell a related story about difficulties in maintaining farm hygiene practice. For groups with little ‘spare’ time, such as the urban poor, we need to find suitable time for events that do not interfere with their working schedules. In low and middle- income countries, people who are less well-off have to use government hospitals which are cheaper but often with long waiting times. It was suggested that this waiting time would be a potential time to engage them with PE activities. We can approach them for interviews or put up a Q&A box or a board where people can write their own questions and concerns.

### Integration into local lives

Disadvantaged populations may be occupied with earning a living and it is often reported that they pay less attention to healthcare, science or research ( Cortis, 2012; Freimuth & Mettger, 1990; Hanafin & Lynch, 2002). Difference in culture and historical events may also make them resistant to interventions from outsiders. Therefore, one key solution to reach these populations is to integrate engagement activities into their daily lives and common practices.

For the urban poor, the waiting lounge at public health institutions can be used to display videos or distribute leaflets. We can also utilize break time to deliver any engagement activities so as not to interfere with their busy pace of life. For example, in Hospital for Tropical Diseases in Vietnam, the OUCRU PE team are conducting a radio show in the early evenings when medical procedures have finished, with the aim of providing health education to patients and their families in the hospital. For workers in industrial zones, science cafés or health talks could be conducted during lunchtimes or at the weekends so working time of employees is not affected. MORU Science Café events are usually held in the hospital grounds and village coffee shops to engage the public to discuss about biomedical science topics ( Cheah *et al.*, 2016; Pol *et al*., 2017).

To prevent overwhelming the school community, engagement with rural school children was suggested to either become part of their official school activities or as a one-off event at weekends and mid-term breaks. One example is the Science Theatre shows or Science Festival days held by the OUCRU PE team, which are held early in the morning before school starts, or during the lunch breaks.

For ethnic minority communities, we suggested that activities should be embedded within their own culture to induce the community’s interest to learn about health and science. Activities can take place during local festivals and gatherings such as community meetings. We can also integrate the education component into their local daily activities such as art performances, cultural games, paintings for children, embroidery for women, and so on. It was also emphasized to let the community take the artist role, in which they tell their own stories under guidance of experts. For example, the Village Drama Against Malaria project in Cambodia has successfully organized local theatre plays about malaria performed by local children with support from scientists and professional artists in 20 remote villages (Lim *et al.*, 2016).

### Networking with relevant stakeholders

Collaboration with key local contacts is considered an important impact factor on the outcome of engagement programme for the disadvantaged population ( Abrams, 2010; Bonevski *et al.*, 2014; Cortis, 2012). Our workshop participants proposed a wide variety of stakeholders ranging from the staff that may directly be involve in delivering engagement activities such as healthcare workers, teachers, and scientists, to people with higher power and social levels such as the local authorities, school and factory managers, religious leaders, village leaders or the elderly, and partners that potentially benefits our outreach.

Local staff and agencies are an important factor in smoothing interactions and promoting participation with the communities as they are familiar with and have the trust of local people. For the urban poor, we can cooperate with local social and non-profit organizations. In Thailand, there are many community-based organisations that have long-established work with the street communities while in Vietnam urban areas, there are voluntary groups who organizing support classes and charitable activities for homeless or disadvantaged children. For ethnic minorities, local gatekeepers such as village and religious leaders, elders, or representatives from local administration groups like Youth Union staff or a Women’s Union officer can be very helpful in encouraging participation. This is particularly beneficial in conducting biomedical research. The Tak Province Community Ethics Advisory Board in Maesot, Thailand, have been in operation for more than ten years, inviting key stakeholders from the research community to regular meetings to give advice and comments for clinical studies (Lwin *et al.*, 2014). For the rural children, a feasible, and arguably more sustainable, approach is to contact schools and let teachers introduce and conduct activities. In many remote areas, teachers are highly-respected among the community and able to positively influence the children and their parents.

Although sometimes it comes with heavier bureaucracy, partnerships with people in power such as the local authorities can be beneficial for implementation of the engagement activities and ensures that they comply with national policies and regulations. Moreover, the engagement programmes can have the potential to impact a wider audience through influencing local policies. For example, by working closely with the provincial-level Department of Education in Vietnam, OUCRU PE department was able to positively influence the inclusion of sex and relationship training for teachers. This would not have been possible by working directly with each school.

### Developing local resources

PE practitioners are paying more attention to the sustainability of activities by enhancing both human and physical resources in the local areas. Many engagement programmes now include a capacity-building component to maintain sustainability of activities. Enhancing local human resources aim to empower the community by equipping them with adequate knowledge and skills and improving their motivation to continue engagement activities. In Maesot, Thailand, the Shoklo Malaria Research Unit has set up clinics inside the community and trained ethnic nurses to deliver healthcare for the local residents. To engage the primary school communities in rural schools in Ben Tre, Vietnam, teachers are trained to deliver participatory science activities such as simple experiments during lessons and organize science clubs as extra-curricular activities.

Due to the shortage in modern facilities and equipment in disadvantaged areas, our participants proposed that instead of introducing complicated technology, we can provide the local community with simple creative resources. For example, we can distribute simple media such as leaflets and handbooks that attract children’s interest. Although we have to take care not to offer undue incentives, we can also partner with other groups (local charities etc), to offer teaching resources such as books, furniture, and equipment.

## Conclusions

Existing literature reflects an increasing need for more efforts towards PE targeting the underserved population. Discussion of participants from low and middle-income settings in this regional workshop presented three hard-to-reach groups with both similar and distinctive characteristics that make them susceptible to exclusion from mainstream intervention. Barriers to reach them result from difficulties in a wide range of financial, cultural, and structural factors at both individual and community levels. To overcome these challenges, our workshop participants highlighted the importance of a meaningful and genuine understanding of the target population as well as a comprehensive approach to facilitate sustainable changes. We came up with strategies that can be summarized by the FIND acronym: Formative research to meaningfully explore the context, Integration into local lives, Networking with relevant stakeholders, and developing local resources, aiming at creating activities that can become a normal practice in the life of the local communities.

## Strengths and limitations

The strength of this conference discussion is that the delegates were mainly Southeast Asian nationals (36/38 people) and have had years of experience of working in a wide range of engagement and development projects, so we were able to draw ideas from multiple perspectives and experiences. Our ideas may be useful for adaptation to engagement activities in similar contexts.

However, we acknowledge that this is not an exhaustive list of hard-to-reach groups in Southeast Asia, but are the communities that our group of engagement practitioners are aware of and interested to reach. We are a relatively small group and there was a limited time to discuss into specific details of each hard-to-reach population.

## Data availability

No data are associated with this article.
