# Supplementary material for: Identifying ‘hard-to-reach’ groups and strategies to engage them in biomedical research: perspectives from engagement practitioners in Southeast Asia
Source: Wellcome Open Res. 2019 Jun 26;4:102. [Version 1] doi: 10.12688/wellcomeopenres.15326.1 (PMC6823897; doi:10.12688/wellcomeopenres.15326.1)
Supplement: Peer review history

# Peer review: Reviewer response for version 1

**DOI:** 10.21956/wellcomeopenres.16731.r36729

**Published:** 2019-10-31

**Authors:** Paul Murphy

**Affiliations:** 1 School of Arts, English and Languages, Queen's University Belfast, Belfast, UK

This article constitutes a report on a workshop involving public engagement (PE) practitioners from across Southeast Asia that engaged in a discussion about ‘hard-to-reach’ populations in the region and how PE can better engage them. The article is well written and the analysis of PE is situated in relation to relevant literature in the field of enquiry.

 The nuances of ‘hard-to-reach’ as both an epistemological and ontological category are discussed apropos of broader contingencies such as financial instability, mobility in residence and work, social discrimination and isolation, and limitations in local resources.

 The ‘FIND’ framework developed in the workshop is interesting not least in terms of the geographical context discussed in the report but moreover for its potential relevance and applicability to contexts across the world. The strategies contained in the FIND framework may, at first glance, seem common sense approaches to the subject matter when viewed in isolation, but when considered as a holistic framework the strategies offer added value by virtue of their systematic integration.

 There is a strong ethnographic tendency in the framework that could have been highlighted more explicitly and discussed in relation to contemporary debates in ethnography. For example, the issue of ethnic minorities being ‘reserved in talking to the “outsiders”’ is highlighted in the article and is well documented in ethnographic research. The emphasis on ‘understanding the local life’ and avoiding ‘misconceptions’ is a key consideration in ethnography that could be enhanced by engagement with research in this field. The FIND framework could perhaps be enhanced by considering these suggestions to develop and refine a promising PE strategy.

I confirm that I have read this submission and believe that I have an appropriate level of expertise to confirm that it is of an acceptable scientific standard.

# Peer review: Reviewer response for version 1

**DOI:** 10.21956/wellcomeopenres.16731.r36539

**Published:** 2019-10-09

**Authors:** Bella Starling

**Affiliations:** 1 Public Programmes Team, Research and Innovation Division, Manchester University NHS Foundation Trust (MFT), Manchester, UK

This is a report of a workshop held by practitioners of public engagement, mainly based in South East Asia. The report focuses on the barriers to engaging seldom heard groups with biomedical research, and offers a light-tough framework and practical tips to work with these groups. 

 It is accessibly written, provides practical grounding and guidance illustrated with really clear and relevant examples, and is adequately referenced for a report of this length and format. 

 It draws a good balance between the financial, cultural and structural factors that can exclude marginalised people from research and engagement. Although it is touched on, perhaps the discussion could have gone deeper into the research culture change needed to better support more inclusive engagement, moving the onus of responsibility away from those already marginalised and from public engagement practitioners themselves already working with limited resources. 

 The report is useful in elaborating the context of so-called 'harder to reach' communities in low to middle income countries, and how these contexts might differ from public engagement practice and settings in the global North. This reviewer would argue that there are some (but not all) transferable lessons between the two contexts, and the report could highlight some of these. For example, the FIND framework could also be useful to practitioners elsewhere. Although it is implicit, the framework could more explicitly articulate the need to listen to stakeholders, not just in terms of understanding contexts, but also with respect to influencing methods of engagement and the conduct of research. 

 Although it's outwith the scope of this article, it would be interesting to know whether any discussions at the workshop covered tensions or complementarities (perceived or real) between recruitment to research studies, and engagement with research. 

 I really enjoyed reading the article. Thank you to the authors.

I confirm that I have read this submission and believe that I have an appropriate level of expertise to confirm that it is of an acceptable scientific standard.
